# Lethal metabolism of *Candida albicans* respiratory mutants

**DOI:** 10.1371/journal.pone.0300630

**Published:** 2024-04-05

**Authors:** D. Lucas Kane, Brendan Burke, Monica Diaz, Christian Wolf, William A. Fonzi

**Affiliations:** 1 Department of Chemistry and Medicinal Chemistry Shared Resource, Georgetown University, Washington, DC, United States of America; 2 Department of Microbiology, Georgetown University, Washington, DC, United States of America; University of Nevada Reno, UNITED STATES

## Abstract

The destructive impact of fungi in agriculture and animal and human health, coincident with increases in antifungal resistance, underscores the need for new and alternative drug targets to counteract these trends. Cellular metabolism relies on many intermediates with intrinsic toxicity and promiscuous enzymatic activity generates others. Fuller knowledge of these toxic entities and their generation may offer opportunities of antifungal development. From this perspective our observation of media-conditional lethal metabolism in respiratory mutants of the opportunistic fungal pathogen *Candida albicans* was of interest. *C*. *albicans* mutants defective in NADH:ubiquinone oxidoreductase (Complex I of the electron transport chain) exhibit normal growth in synthetic complete medium. In YPD medium, however, the mutants grow normally until early stationary phase whereupon a dramatic loss of viability occurs. Upwards of 90% of cells die over the subsequent four to six hours with a loss of membrane integrity. The extent of cell death was proportional to the amount of BactoPeptone, and to a lesser extent, the amount of yeast extract. YPD medium conditioned by growth of the mutant was toxic to wild-type cells indicating mutant metabolism established a toxic milieu in the media. Conditioned media contained a volatile component that contributed to toxicity, but only in the presence of a component of BactoPeptone. Fractionation experiments revealed purine nucleosides or bases as the synergistic component. GC-mass spectrometry analysis revealed acetal (1,1-diethoxyethane) as the active volatile. This previously unreported and lethal synergistic interaction of acetal and purines suggests a hitherto unrecognized toxic metabolism potentially exploitable in the search for antifungal targets.

## Introduction

The destructive impact of fungi in agriculture and animal and human health is increasing due to multiple anthropic factors [1]. Coincident increases in resistance to antifungal agents further exacerbates concerns for protection of world food supplies and clinical management of fungal infections [2]. Recent revelations of fungal association with various cancers raises further concerns [3–5]. The magnitude of morbidity and mortality associated with human fungal infections, while difficult to quantify precisely, is nonetheless, vast, e.g. 1,000,000 cases/year of cryptococcosis, 400,000/year of candidiasis and pneumocystis and 200,000/year of aspergillosis [6]. This translates into an economic burden of about $11.5 billion in the US alone [7]. Disturbing as these numbers may be, the inadequacy of current therapeutic interventions is more so. Mortality associated with these infections ranges from 20 to 95% despite treatment [6]. The need for new and more efficacious antifungals is obvious, and while progress is being made in identifying new drugs and drug targets, the need for new and alternative targets and approaches will not dissipate [8].

A more complete mapping of cellular metabolism may prove useful. Cellular metabolism, though essential to life, inescapably results in production of intermediate metabolites, some of which have toxic effects if permitted to accumulate. Leveraging this toxicity may lead to development of new antimicrobials and anticancer drugs [9, 10]. The potential of this approach has been demonstrated in a series of elegant studies of fructose-asparagine (F-Asn) metabolism in *Salmonella enterica* Typhimurium. F-Asn is found in a number of foods and can be used as a carbon and nitrogen source by *S*. Typhimurium [11, 12]. Catabolism of F-Asn proceeds through deamination to fructose-aspartate which is phosphorylation to produce 6-phosphofructose-aspartate [13]. The final step, catalyzed by the *fraB* encoded glycanase, yields glucose-6-phosphate and aspartate [13]. In the absence of *fraB*, 6-phosphofructose-aspartate accumulates and attenuates growth and virulence [11, 13]. Several small molecule inhibitors of the *fraB* glycanase were recently identified as potential narrow spectrum antimicrobials [9]. This approach was recently expanded to consider other toxic sugar-phosphate intermediates [14, 15].

Aside from sugar-phosphates, many other intermediate metabolites are toxic and, therefore potential pressure points. Furthermore, the true number of toxic metabolites is likely underappreciated. Examples of known toxic metabolites include homoserine [16], the acrylyl-CoA intermediate in propionyl-CoA synthesis [17, 18], propionyl-CoA itself [19], methylmalonic acid [20], 5′-methylthioadenosine [21], succinic semialdehyde [22], glutarate [23], nicotinate and diacylglycerol [24], to name a few. Toxicity arises from their ability to inhibit critical enzymes or their inherent chemical reactivity [25]. In addition to known canonical metabolites there may be a plethora of non-canonical metabolites. Untargeted metabolomics has revealed far more metabolites than accounted for by known metabolic pathways, 90% or more of which are unidentified, so-called dark molecules [26]. Their genesis may lie in substrate or catalytic promiscuity of enzymes [27] or arise as a consequence of the inherent chemical reactivity of many metabolites [25]. These non-canonical metabolites are of serious consequence to the cell as evidenced by the expanding catalog of “metabolite repair” and preemptive pathways that metabolize them [28, 29].

It was from this perspective that we initiated investigation of a unique form of media-conditional lethal metabolism in respiratory mutants of the opportunistic fungal pathogen *Candida albicans*. When cultured in synthetic complete medium, mutants lacking NADH:ubiquinone oxidoreductase (Complex I of the electron transport chain) exhibit normal growth and morphology. However, when cultured in yeast extract-peptone-dextrose medium (YPD), the mutants grow normally until early stationary phase whereupon a dramatic loss of viability occurs. Upwards of 90% of cells die over the subsequent four to six hours with a loss of membrane integrity. The extent of cell death was proportional to the amount of BactoPeptone added to the medium, and to a lesser extent, the amount of yeast extract added. YPD medium conditioned by growth of the mutant was toxic to wild-type cells indicating mutant metabolism established a toxic milieu in the media. Conditioned media contained a volatile component that contributed to toxicity, but only in the presence of a component of BactoPeptone. Fractionation experiments revealed purine nucleosides or bases as the synergistic component. GC-mass spectrometry analysis revealed acetal (1,1-diethoxyethane) (henceforth referred to as acetal) as the active volatile. This previously unreported and lethal synergistic interaction of acetal and purines suggests a heretofore unrecognized toxic metabolism potentially exploitable in the search for antifungal targets.

## Materials and methods

### Strains, media and culture conditions

The strains used are listed in Table 1. YPD medium consisted of 2% glucose, 2% BactoPeptone (Gibco) and 1% yeast extract (Gibco). SC medium consisted of 2% glucose, 1X Drop-out Mix Synthetic minus uracil, without yeast nitrogen base (USBiological Life Sciences), 0.5% ammonium sulfate, 1X BD Difco Yeast Nitrogen Base without ammonium sulfate and amino acids, and 100 μg/ml uridine. SC medium was adjusted to pH 6.6 to 6.8 except were indicated otherwise. CFU determinations were performed using SC medium solidified with 2% agar.

**Table 1:** Strains used.

Strain	Genotype	Reference
SC5314	wild-type	[31]
mt1179.41	*nuo3*Δ::Cm*LEU2*/*nuo3*Δ::Cd*HIS1 leu2*Δ/*leu2*Δ *his1*Δ/*his1*Δ *ARG4*/*arg4*Δ	[32]
	*URA3*/*ura3*Δ::*imm*^*434*^ *IRO1*/*iro1*Δ::*imm*^*434*^	
mt5077.41	*nuo4*Δ::Cm*LEU2*/*nuo4*Δ::Cd*HIS1 leu2*Δ/*leu2*Δ *his1*Δ/*his1*Δ *ARG4*/*arg4*Δ	[32]
	*URA3*/*ura3*Δ::*imm*^*434*^ *IRO1*/*iro1*Δ::*imm*^*434*^	
mt2819.41	*nue1*Δ::Cm*LEU2*/*nue1*Δ::Cd*HIS1 leu2*Δ/*leu2*Δ *his1*Δ/*his1*Δ *ARG4*/*arg4*Δ	[32]
	*URA3*/*ura3*Δ::*imm*^*434*^ *IRO1*/*iro1*Δ::*imm*^*434*^	
mt4467.41	*nue2*Δ::Cm*LEU2*/*nue2*Δ::Cd*HIS1 leu2*Δ/*leu2*Δ *his1*Δ/*his1*Δ *ARG4*/*arg4*Δ	[32]
	*URA3*/*ura3*Δ::*imm*^*434*^ *IRO1*/*iro1*Δ::*imm*^*434*^	
mt2513.41	*mne1Δ*::*CmLEU2/mne1Δ*::*CdHIS1 ARG4/arg4Δ*	[32]
	*leu2Δ/leu2Δ his1Δ/his1Δ URA3/ura3Δ*::*imm434 IRO1/iro1Δ*::*imm434*	
GOA1ms.41	*goa1Δ*::*CmLEU2/goa1Δ*::*CdHIS1 leu2Δ/leu2Δ his1Δ/his1Δ ARG4/arg4Δ*	[32]
	*URA3/ura3Δ*::*imm434 IRO1/iro1Δ*::*imm434*	

For media-conditional tests, strains were inoculated into SC medium from stock cultures stored at -80°C and incubated 24 h at 30°C. Cell density was determined by hemocytometer count and cells inoculated at an initial density of 1 x 10^5^ cells/ml in 10 ml of test media in 125 ml flasks with foil caps incubated at 30°C in a rotary water bath shaking at approximately 250 RPM. After 24 h, cell viability was assessed by trypan blue staining.

SC5314 cells for toxicity tests were cultured in YPD. Log-phase cells were collected by centrifugation at a density of 1 x 10^7^ cells/ml, stationary-phase cells at a density of 3 x 10^8^ cells/ml. Cells were washed once with 1/10^th^ volume of PBS and suspended in fresh PBS prior to inoculation of test media. Test media (10 ml) was inoculated to an initial cell density of 5 X 10^6^ cells/ml and incubated as above.

### Conditioned media preparation and purging

Preparation of conditioned media was initiated by inoculating 250 ml of media in a 1 l flask to a density of 1 x 10^5^ cells/ml. Cultures were incubated at 30°C and shaken at 250 RPM until a density of approximately 1 x 10^8^ cells/ml, the cell density attained just prior to the onset of cell death as determined by time course studies. The cultures were then centrifuged 10 min at 4,000 x g, 4°C. The supernatant solution was collected, filtered sterilized and stored at 4°C until use.

Purged media was prepared by placing 100 ml conditioned media in a 500 ml flask, warmed to 40°C in a water bath and bubbling sterile air into the media at a flow rate of 1 l/h while mixing with a magnetic stir bar. The media was purged for 2.5 h then stored at -20°C till use.

### Fractionation of BactoPeptone

A 1.5 x 50 cm column of Bio-Gel P2 (extra fine) equilibrated with 20 mM ammonium acetate, pH 8.2, was loaded with 1 ml 20% (w/v) BactoPeptone and eluted at a flow rate of 0.22 ml/min. Fractions of 1.5 ml were collected, transferred to a 2 ml plastic microfuge tube, frozen and vacuum dried in a SpeedVac at room temperature. Activity of the fractions was assessed following dissolution in 0.5 ml of mt1179.41 (*nuo3Δ/Δ*)-conditioned SC media containing 5 x 10^6^ cells/ml of log-phase SC5314 cells and incubation for approximately 16 h at 30°C on a tube rotator.

### Capture of volatiles

The volatile components of conditioned media were captured by condensation or adsorption. Condensate preparation was initiated by inoculating 1 l SC media in a 2 l Erlenmeyer flask at a density of 1 x 10^5^ cells/ml and incubating in a shaking water bath at 30°C, 250 RPM. When the culture reached a density of 1 x 10^8^ cells/ml a plug containing inlet and outlet tubes was inserted and sterile air was bubbled into the media at a flow rate of 1 l/min with continuous shaking. The exhaust air was directed into a Dewar’s type vacuum trap cooled with dry ice–ethanol. After 6.5 h, condensate in the trap was thawed, collected and stored at -20°C until use. The yield was approximately 3 ml.

Adsorption capture was conducted using 2 ml glass Pasteur pipettes plugged with glass wool and packed with 25 mg of activated charcoal. The charcoal was rinsed with 2 ml dichloromethane and vacuum dried prior to use. Conditioned SC medium, 200 ml in a 1 l flask, was heated to 40°C in a water bath, mixed with a magnetic stir bar and sterile air was bubbled though the media at a flow rate of 50 ml/min. The exhaust was passed via Tygon tubing through the charcoal trap housed in an oven at 50°C. After 2.5 h, the pipet was removed and stored in a sealed glass tube at -20°C until analysis.

### GC-MS analysis of extracts obtained from aqueous solutions and charcoal materials

Dichloromethane was purchased from Fisher Scientific and used without further purification. Cyclohexene served as internal reference. None of the volatile analytes observed in this study were present in dichloromethane blanks. GC-MS measurements were performed on an Agilent 5977C GC/MSD equipped with an HP-5ms Ultra Inert (5%-phenyl)methylpolysiloxane column (30 m, 0.25 mm, 0.25 μm). Gas chromatograms were collected with 3.0 microliter injection volumes, at 50°C, 1.2 ml/min He carrier gas flow, and a 9.8 psi setpoint pressure. Mass spectra were obtained using electron-impact ionization and cross-referenced with the NIST MS database for structural assignment.

Extraction from aqueous solution: Portions of 0.5 ml of aqueous condensate solution from either the wild-type or mutant cultures were placed into an 8 ml vial, along with 0.5 ml of dichloromethane. The vial was sealed, and the mixture was thoroughly agitated. Once settled, the organic layer was collected and transferred to a vial for GC-MS analysis.

Extraction from charcoal plugs: The Pasteur pipettes containing the charcoal-bound analytes from either the wild-type or mutant cultures were rinsed directly with 1.50 ml of dichloromethane to elute the organic material from the charcoal. The resulting solution was transferred into a vial for GC-MS analysis.

### Trypan blue assay

At indicated time points 0.5 ml samples were removed from cultures and passed through a 1ml insulin syringe with a 30G needle to disaggregrate cell clumps. A 10 μl aliquot was mixed with 190 μl of 0.22% trypan blue in saline and used to load a Neubauer hemocytometer. After cell settling, 150–400 total cells were counted and scored for trypan blue staining to calculate total cell density and dead cell density.

### Ethanol determination

Ethanol concentrations were determine using a coupled enzyme assay (Megazyme) according to the manufacturer’s instructions. Conditioned media was prepared from SC and YPD cultures of strain mt1179.41 or the wild-type strain SC5314 as described above. A portion of each was purged as described above. A 39 μl sample was assayed in a total volume of 1.0 ml containing the supplied buffer and NAD^+^. Aldehyde dehydrogenase was added and absorbance at 340 nm was followed for two min prior to addition of alchohol dehydrogenase. Absorbance 340 was followed for six min to ensure the reaction went to completion. Samples were diluted as needed to achieve a final OD_340_ of approximately 0.5.

## Results

### Media-conditional cell death

**Fig 1:**
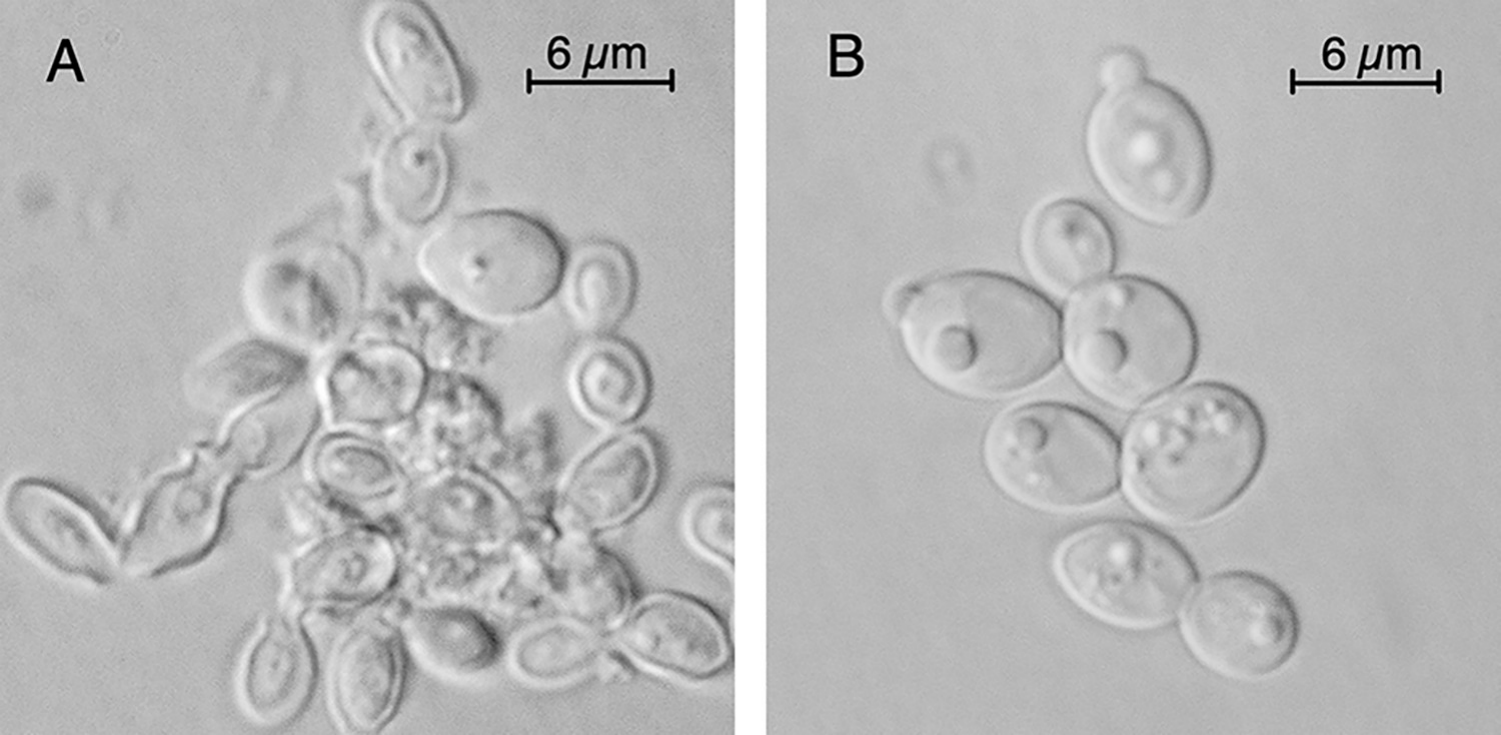
Media-conditional morphology of NADH:ubiquinone oxidoreductase (Complex I) deficient mutant. Cells of strain mt1179.41 (*nuo3Δ/Δ*) cultured to stationary phase in YPD medium (Panel A) or SC medium (Panel B) imaged at 100X magnification using Hoffman modulation contrast optics.

*Candida albicans* contains multiple lineage-specific genes encoding functions required for assembly of a fully operational electron transport chain [30]. These genes participate in the assembly and structure of Complex I, III and IV, NADH ubiquinone oxidoreductase, coenzymeQ:cytochrome c oxidoreductase and cytochrome c oxidase respectively. One of these genes, *NUO3* (orf 19.1179), encodes a subunit of Complex I [30]. The absence of *NUO3* results in a loss of Complex I and a 70% reduction in respiration [30]. While examining the growth behavior of strain mt1179.41, a *nuo3Δ/Δ* null mutant, it was noted that the cells of stationary-phase cultures grown in YPD were agglomerated, many with condensed or apparently extruded cytoplasmic contents (Fig 1). In contrast, cells cultured in SC medium were normal in appearance (Fig 1).

**Fig 2:**
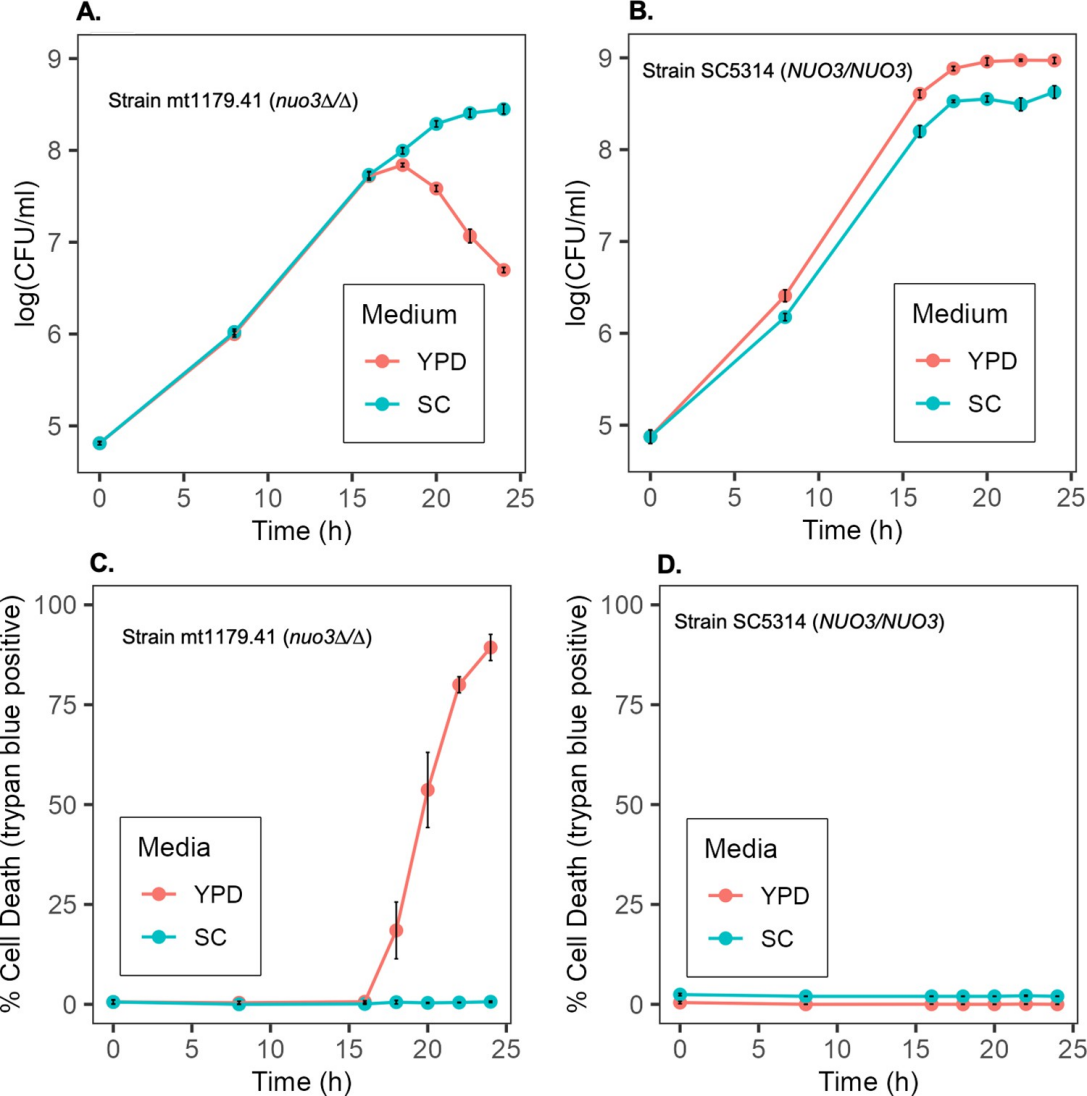
Media-conditional loss of cell viability. Strain mt1179.41 (*nuo3Δ/Δ*) or wild-type strain SC5314 were cultured 24 h to stationary phase in SC medium, inoculated into SC or YPD medium at a density of 1 x 10^5^ cells/ml and sampled at the indicated time points. Samples were plated on SC agar to determine CFU (Panels A and B) or mixed with trypan blue and enumerated microscopically to determine the number of live and dead cells (Panels C and D). Note: the values for SC medium in Panel D are offset by 2% to avoid overlapping lines. Points represent the average and cross-bars indicate standard error of the mean of three independent determinations.

Further examination of the growth behavior of the *nuo3Δ/Δ* mutant revealed a dramatic media-conditional loss of cell viability. Growth of the mutant in SC vs YPD medium was comparable in yield and rate until early stationary phase, a cell density of approximately 1 x 10^8^ cells/ml. At this point there was a striking loss of cell viability in the YPD culture with about 90% of the cells dying over the next few hours (Fig 2). In contrast, viability was fully maintained in SC media. A wild-type control strain, SC5314, retained full viability in both growth media. While plating assays demonstrated the loss of CFU, vital staining with the membrane impermeable dye trypan blue showed that loss of CFU was paralleled by loss of membrane integrity (Fig 2).

**Fig 3:**
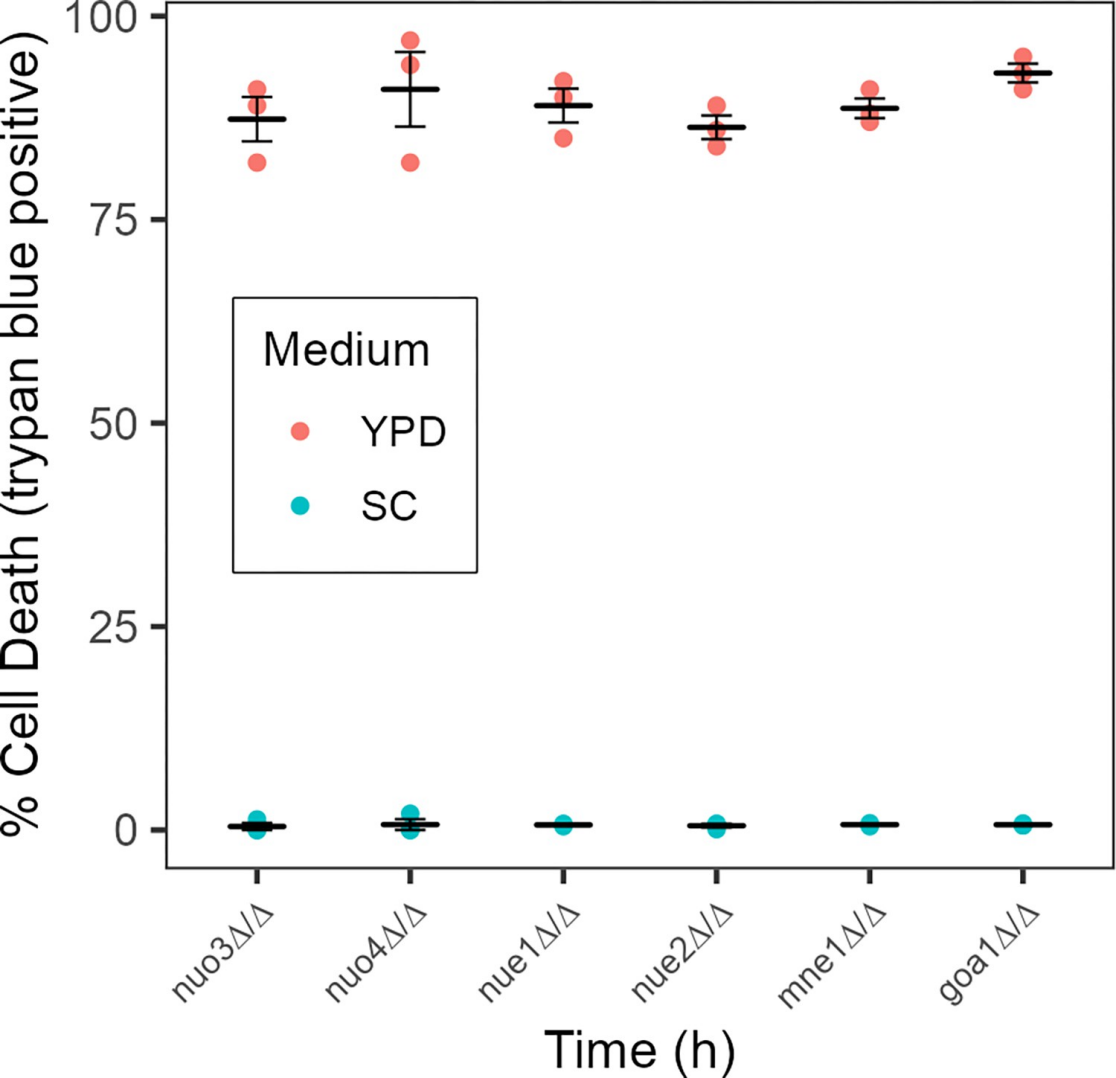
Media conditional viability of Complex I mutants. Deletion mutants lacking genes encoding Complex I subunits (*NUO3* and *NUO4*) or processing/assembly functions (*NUE1*, *NUE2*, *MNE1* and *GOA1*) [32] were inoculated into SC or YPD medium at a density of 1 x 10^5^ cells/ml. After 24 h, cell viability was assessed using trypan blue. Each point indicates an independent determination. Crossbars indicate the average and standard error of the mean of three independent determinations.

This phenotype was not unique to the *nuo3Δ/Δ* mutant. Five additional mutants defective in Complex I were examined (Table 1). These included mutants lacking *NUO4*, another structural subunit of Complex I, and genes involved in Complex I synthesis or assembly, *NUE1*, *NUE2*, *GOA1* and *MNE1* [30]. A similar extent of media conditional cell death occurred in each mutant (Fig 3) indicating that the phenotype was due to the loss of Complex I, not a specific component or process in its production.

### BactoPeptone and yeast extract induce cell death

**Fig 4:**
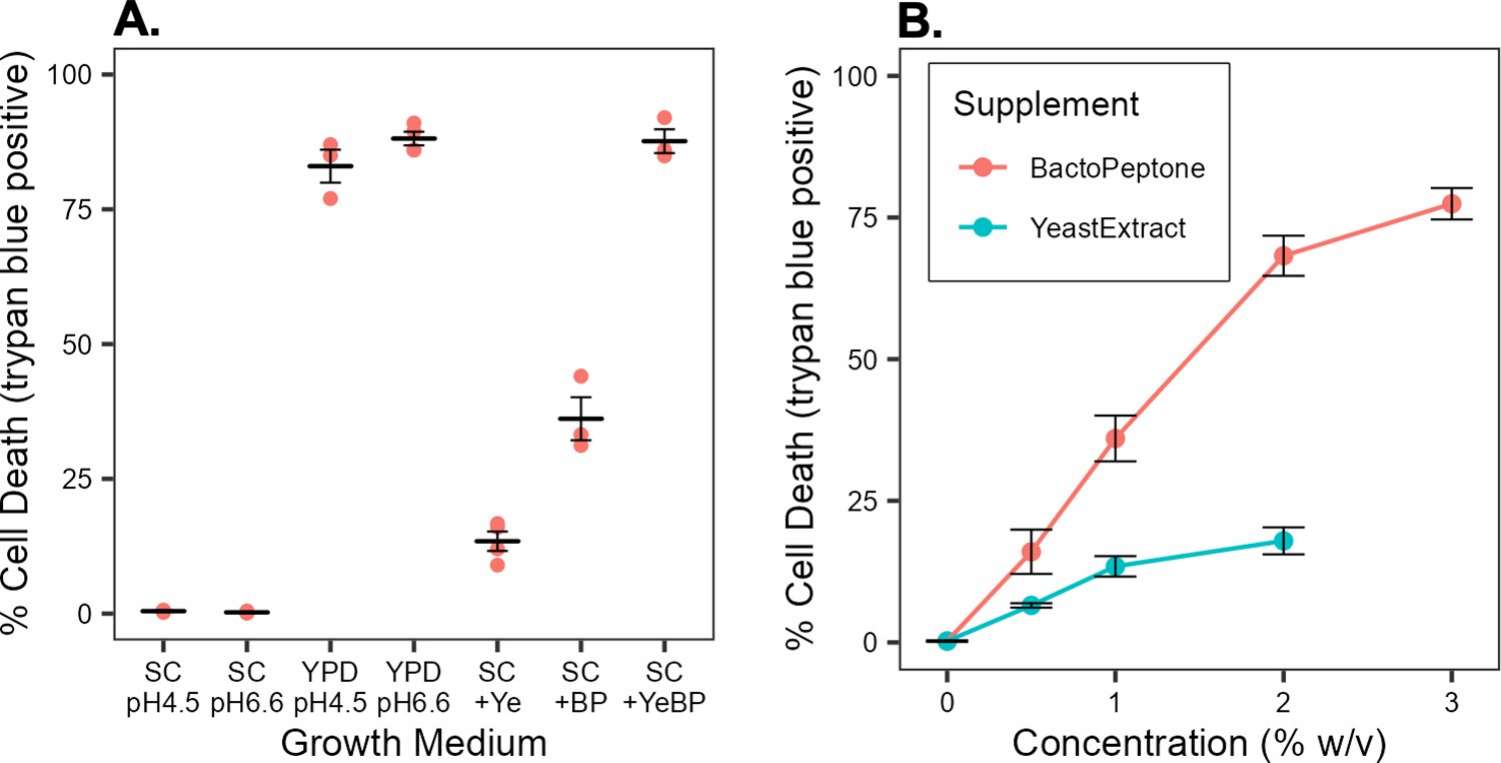
Media factors promoting cell death. Panel A. SC and YPD media were modified in pH or SC medium was supplemented with BactoPeptone or yeast extract prior to culturing strain mt1179.41 (*nuo3Δ/Δ*). Cultures were inoculated with 10^5^ cells/ml of strain mt1179.41 (*nuo3Δ/Δ*). After 24 h, cell viability was assessed using trypan blue. Each point indicates an independent determination. Crossbars indicate the average and standard error of the mean of three independent determinations. Panel B. SC medium was supplemented with various concentrations of BactoPeptone or yeast extract and inoculated with 10^5^ cells/ml of strain mt1179.41 (*nuo3Δ/Δ*). After 24 h, cell viability was assessed using trypan blue. Points represent the average and cross-bars indicate standard error of the mean of three independent determinations.

YPD and SC differ in pH and composition. The conditional phenotype was unrelated to pH differences. YPD has an initial pH near neutrality, pH 6.6 to 6.8, which is largely maintained throughout growth of *C*. *albicans*. In contrast, SC medium has an initial pH of about 4.5 and declines to 3 or less in stationary phase. Adjusting the pH of YPD to pH 4.5 did not prevent loss of viability and, conversely, elevating the pH of SC to 6.6 did not promote cell death (Fig 4). However, addition of BactoPeptone or yeast extract to SC medium induced cell death (Fig 4). Furthermore, there was a positive correlation between the extent of cell death and the amount of BactoPeptone or yeast extract added, Pearson correlation coefficients of 0.96 and 0.94 respectively, with BactoPeptone exerting the greater effect (Fig 4). This was observed with multiple batches of BactoPeptone, as well as Proteose Peptone, but neither Tryptone nor Casamino acids (S1 Table). The results indicated that one or more constituents common to these supplements is deleterious to the mutant.

### The *nuo3Δ/Δ* mutant produces a toxic milieu in the medium

**Fig 5:**
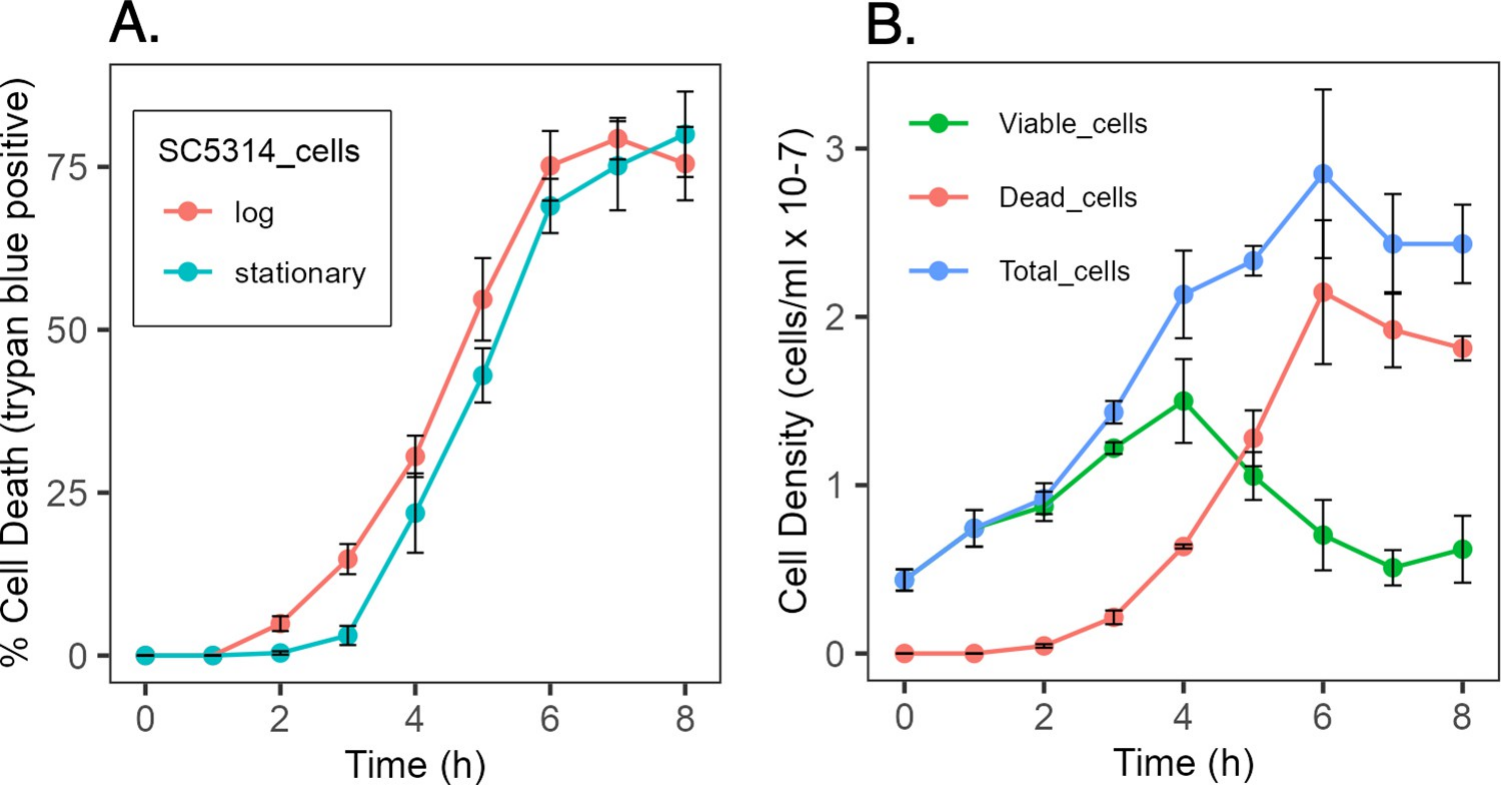
Cell death kinetics in conditioned YPD media. Cells of wild-type strain SC5314 were collected in log or stationary phase and suspended in YPD media conditioned by prior growth of strain mt1179.41 (*nuo3Δ/Δ*) at a density of 5 x 10^6^ cells/ml. Samples were withdrawn at the indicated time points and assessed by trypan blue staining and microscopic count. Panel A displays the percentage of dead cells over time. Panel B displays the cell density of dead, live and total cells. Points represent the average and cross-bars indicate standard error of the mean of three independent determinations.

YPD medium conditioned by growth of the respiratory mutant was toxic to wild-type cells. Conditioned media was prepared by culturing *nuo3Δ/Δ* cells in YPD medium to a density of approximately 1 x 10^8^ cells/ml and removing the cells prior to the onset of cell death. When cells of the wild-type strain, SC5314, were suspended in the conditioned media approximately 80% of the cells lost viability over the next seven to eight hours (Fig 5A). Log-phase and stationary phase cells were equally susceptible, however, the onset of cell death with stationary phase cells was delayed about an hour relative to log-phase cells, consistent with a lag phase and suggesting that toxicity requires actively growing cells (Fig 5A). This was further supported by examination of total cell numbers, which were found to increase prior to the onset of cell death (Fig 5B).

Conditioned YPD medium was similarly toxic to mt1179.41 (*nuo3Δ/Δ*) cells. Mutant cells were cultured to log-phase in SC medium and transferred to conditioned YPD medium. This resulted in a loss of viability comparable in rate and extent to that observed for wild-type cells in conditioned medium (S1 Fig). This suggested both mutant and wild-type cells succumb to the same lethal process. As a control, *nuo3Δ/Δ* cells were subjected to YPD medium conditioned by growth of the wild-type strain. No cell death was observed under these conditions (S1 Fig). Altogether, the results show that the mutant elaborates a compound or compounds toxic to wild-type and mutant cells and that toxicity is not dependent upon respiratory insufficiency.

### Toxicity requires a volatile compound

**Fig 6:**
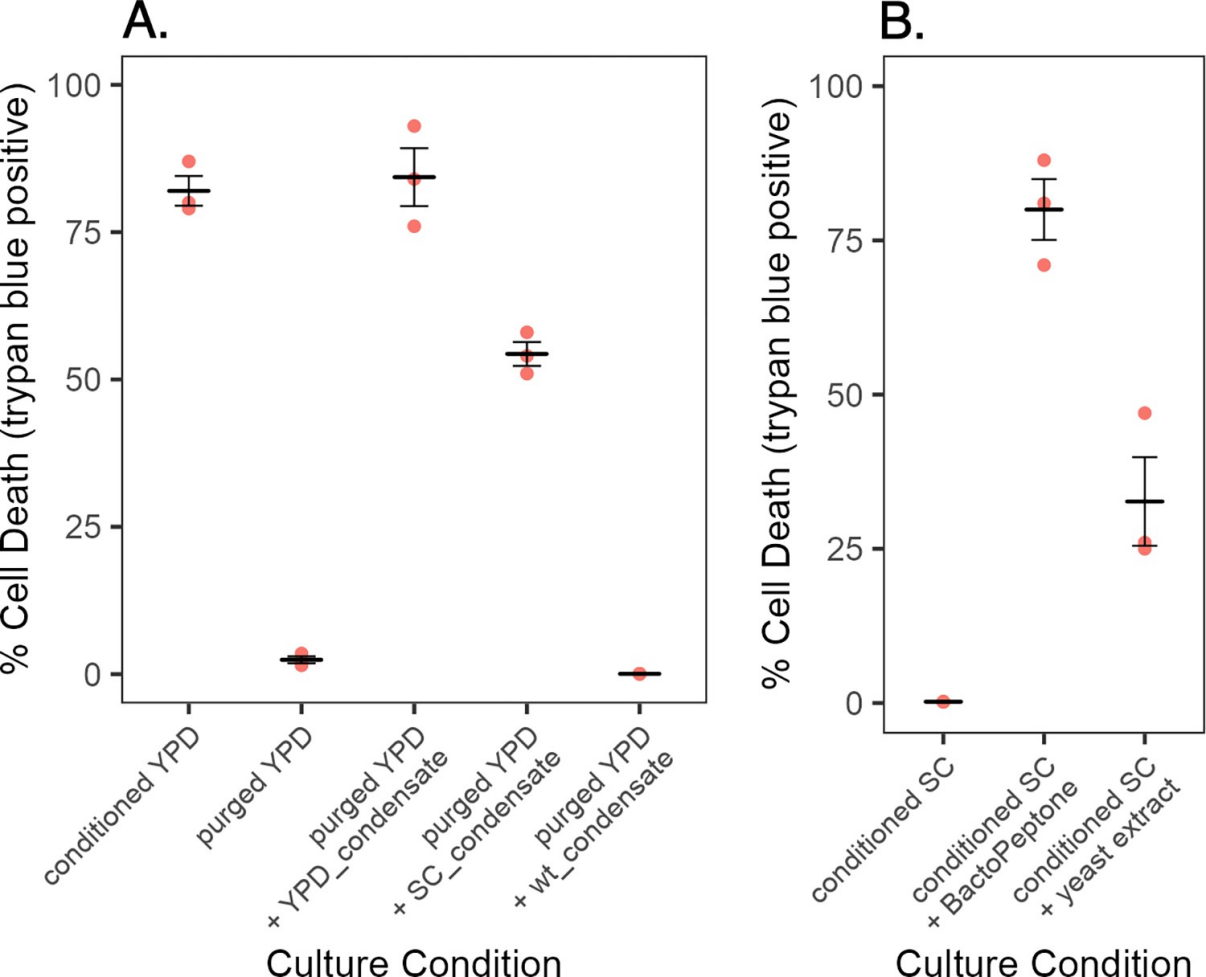
A volatile metabolic product promotes cells death. Panel A. YPD media conditioned by growth of mt1179.41 (*nuo3Δ/Δ*) was unpurged, purged with air, or purged and supplemented with vapor condensate from the indicated purged cultures. Each was inoculated with 5 x 10^6^/ml of wild-type SC5314 cells. After 7 h incubation, viability was assessed by trypan blue staining. Each point indicates an independent determination. Crossbars indicate the average and standard error of the mean of three independent determinations. Panel B. SC media conditioned by growth of mt1179.41 (*nuo3Δ/Δ*) was supplemented with 2% BactoPeptone or 1% yeast extracted. Media were inoculated with log-phase SC5314 cells (5 x 10^6^ cells/ml) and assessed by trypan blue staining after 7 h. Each point indicates an independent determination. Crossbars indicate the average and standard error of the mean of three independent determinations.

Conditioned media contains a volatile component that instigates cell death. Initial attempts at purification of the toxic activity revealed that lyophilization of conditioned media resulted in complete loss of activity indicating the activity was either sensitive to freezing or volatile in nature. These possibilities were distinguished by purging conditioned medium with air and collecting released volatiles by condensation in a cold trap. As shown in Fig 6A, purging conditioned media with air at 40°C removed 97% of the toxic activity. Addition of condensate from the cold trap to the purged media fully restored toxicity. Freezing and thawing conditioned media did not alter its activity. Furthermore, condensate prepared from YPD media conditioned by growth of wild-type cells was without activity (Fig 6A). Thus, mt1179.41 (*nuo3Δ/Δ*) uniquely elaborates a volatile component that promotes cell death.

A quite unexpected result occurred upon testing a control condensate prepared from SC medium conditioned by growth of the mutant. This condensate fully restored toxicity to purged YPD medium (Fig 6A), even though the mutant retains viability in SC medium. This suggested the volatile compound is not intrinsically toxic, but works in conjunction with a component of YPD to promote cell death. This was confirmed when addition of BactoPeptone or yeast extract to conditioned SC medium was shown to promote subsequent cell death (Fig 6B).

### Purine nucleosides and nucleobases promote cell death

The preceding experiment indicated a component of YPD was needed in addition to the volatile compound to initiate cell death. As shown earlier (Fig 3), addition of either yeast extract or BactoPeptone to SC medium stimulated cell death of the *nuo3Δ/Δ* mutant but BactoPeptone had a greater effect. BactoPeptone is a pancreatic digest of meat and offal [33]. The pancreas secretes a broad range of digestive enzymes including proteases, peptidases, lipases, amylase, RNase A and DNase I [34]. Hence, BactoPeptone contains the digestion products of all major cellular macromolecules, as well as cellular metabolites, one or more of which might be relevant to the observed phenotype.

**Fig 7:**
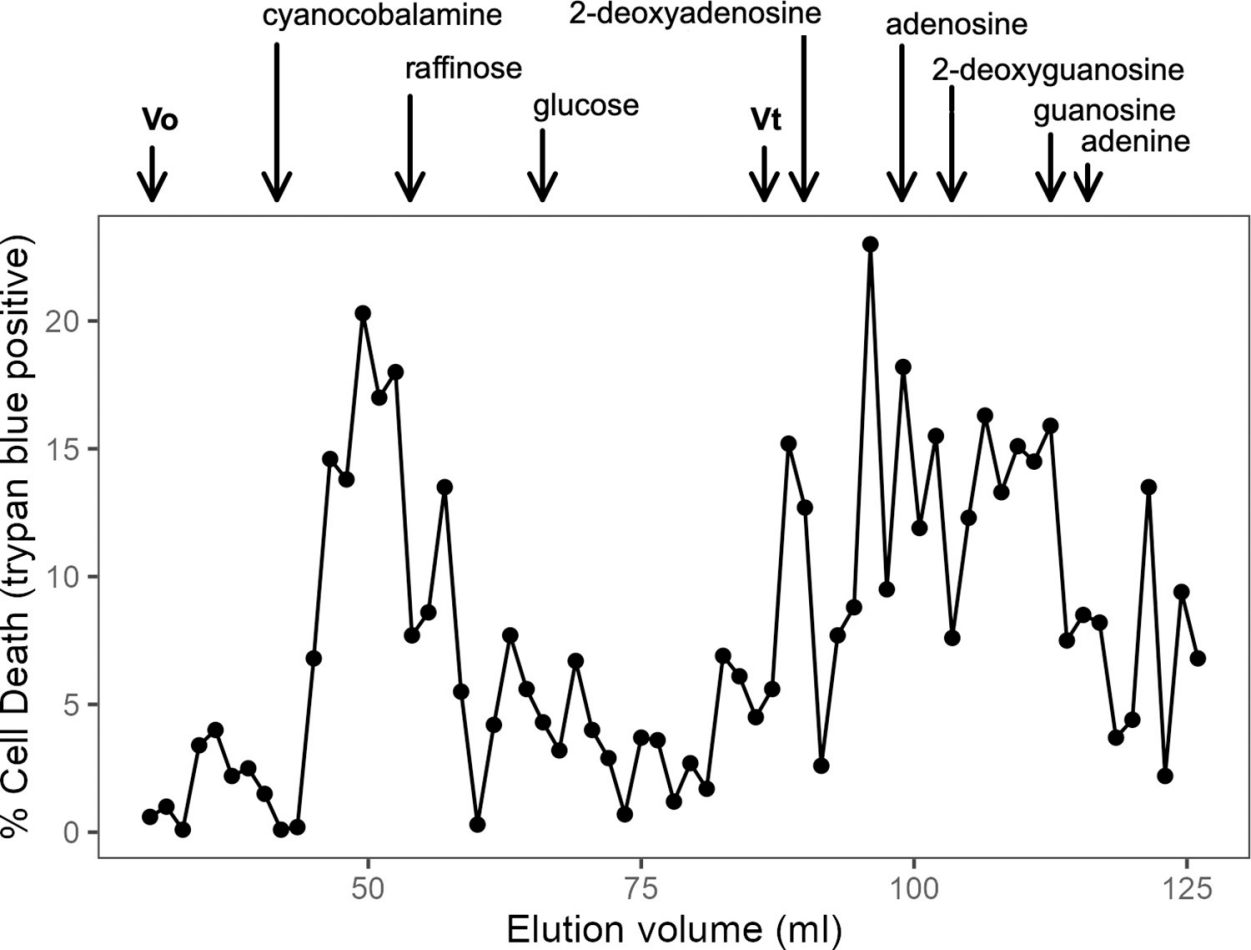
Elution profile of BactoPeptone fractionated on BioGel P2. A 1 ml sample of 20% BactoPeptone in 20 mM ammonium acetate, pH 8.2 was loaded on a 1.5 x 50 cm column and eluted with ammonium acetate buffer. Eluate fractions were dried and dissolved in 0.5 ml of mt1179.41 (*nuo3Δ/Δ*)-conditioned SC medium containing 5 x 10^6^ cells/ml of log-phase SC5314 cells. Viability in each fraction was assessed by trypan blue staining after 16 h incubation. Vo indicates the void volume, Vt indicates the total column volume. The elution position of various test compounds is indicated at the top.

SC medium, like YPD, contains a full complement of amino acids, suggesting amino acids were unlikely to be the relevant component of BactoPeptone. Various lipid extraction procedures failed to remove the stimulating activity from BactoPeptone indicating a hydrophilic component was involved. In an effort to assess the size of the active compound, a BactoPeptone solution was fractionated on a Bio-Rad P-2 gel filtration column and the fractions assessed for their ability to complement the volatile component in cell toxicity. The elution profile (Fig 7) showed multiple fractions with various levels of activity and spread throughout the column. Thus, BactoPeptone contains a number of compounds that stimulate the loss of cell viability. Of particular note was the elution of activity well beyond the total column volume (Fig 7). Delayed elution indicates adsorption of active compounds to the polyacrylamide matrix of P-2, a characteristic of nucleotides and nucleosides and previously used in their purification [35–37].

**Fig 8:**
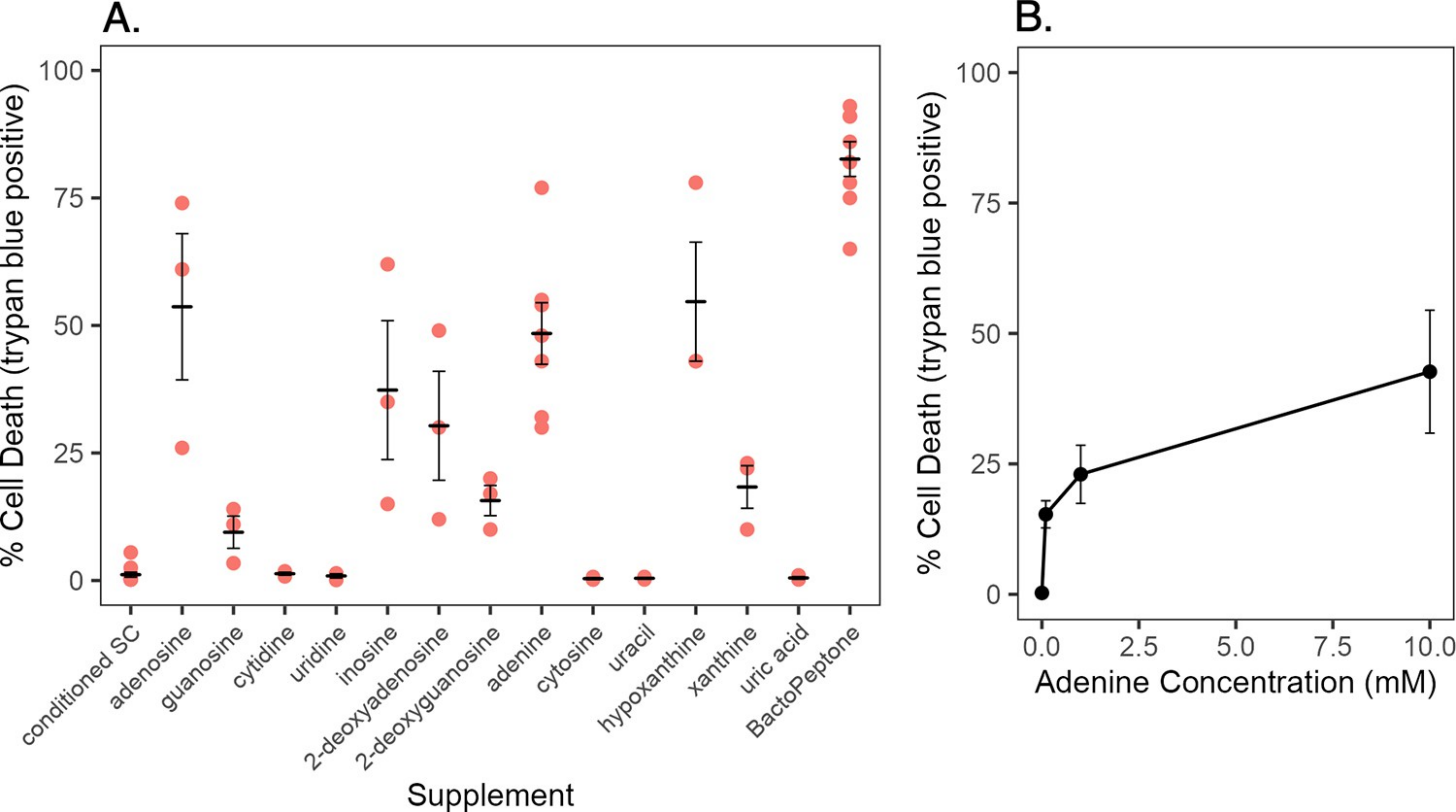
Purines promote cell death. Panel A shows the results of supplementation of mt1179.41(*nuo3Δ/Δ*)-conditioned SC medium with the indicated nucleosides, deoxynucleosides or bases. The final concentration of test compounds was 10 mM, except xanthine and hypoxanthine (5 mM) and guanosine (2.5 mM). Media were inoculated with log-phase SC5314 cells (5 x 10^6^ cells/ml) and assessed by trypan blue staining after 7 h. Each point indicates an independent determination. Crossbars indicate the average and standard error of the mean of at least three independent determinations. Panel B show the effect of varying adenine concentration. Media were inoculated with log-phase SC5314 cells (5 x 10^6^ cells/ml) and assessed by trypan blue staining after 7 h. Each point indicates the average of three independent determinations. Crossbars indicate standard error of the mean of three independent determinations.

Testing of various nucleic acids demonstrated that both ribo- and deoxyribonucleosides containing a purine base, as well as purine bases, stimulated activity. Conditioned SC medium was supplemented with various nucleosides or bases and the effect on wild-type cells examined (Fig 8A). Adenosine, 2-deoxyadenosine and adenine stimulated substantial cell death, as did inosine and hypoxanthine. Lesser, but positive effects, were observed for guanosine, 2-deoxyguanosine and xanthine. In contrast, none of the pyrimidine containing compounds had a detectable effect, including cytidine, cytosine, uridine and uracil. If the conditioned SC medium was purged prior to compound addition, there was no loss of viability (S2 Table). Thus, purines and their nucleic acid derivatives promote cell death in the presence of the volatile compound produced by the *nuo3Δ/Δ* mutant. In addition, there was a positive correlation between purine concentration and the extent of cell death, as seen for BactoPeptone addition (Fig 8B).

As purines can be assimilated via a salvage pathway or catabolized as a nitrogen source, the effect of uric acid was examined. Conversion of xanthine to uric acid via a xanthine hydroxylase is the first step in purine catabolism [38, 39]. If catabolism of purines promoted cell death, addition of uric acid or down-stream metabolites should show a similar effect. However, uric acid supplementation failed to promote cell death (Fig 8A) leading to the conclusion that purine salvage pathways are involved in the toxic effect.

### Identification of volatile organic compounds unique to the *nuo3Δ/Δ* mutant

Given the reduced respiration of the *nuo3Δ/Δ* strain [32] it was likely that aerobic fermentation would be enhanced resulting in elevated ethanol production, a potentially toxic and volatile end product. Indeed, ethanol concentrations measured in *nuo3Δ/Δ* conditioned YPD and SC media were elevated as much as 20-fold over that of media conditioned by wild-type cells (Table 2). However, the concentration was reduced only about 10% by purging of the media and the concentrations were well below those shown to be toxic to *C*. *albicans* [40]. Furthermore, supplementation of YPD medium with a comparable concentration of ethanol did not alter viability of wild-type cells (0.2 ± 0.1% cell death). These results suggested that ethanol was not the volatile of interest.

**Table 2:** Ethanol content of conditioned media.

Strain	Media	Ethanol (mg/ml ± SEM)
mt1179.41	YPD	1.92 ± 0.10
	SC	1.92 ± 0.17
	YPD, purged	1.73 ± 0.15
SC5314	YPD	0.10 ± 0.01
	SC	0.45 ± 0.11

In the organic extracts obtained from the aqueous samples, we found several compounds that were present in both the wild-type and the mutant cultures. 2-Heptenal, 2-cyclohexen-1-ol, 2-methylbutanol, 3-methylbutanol, isobutanol, crotonaldehyde, and propionaldehyde were detected in both samples and in similar amounts. Tetramethylurea was detected in the mutant sample but was absent in the wild-type control.

**Fig 9:**
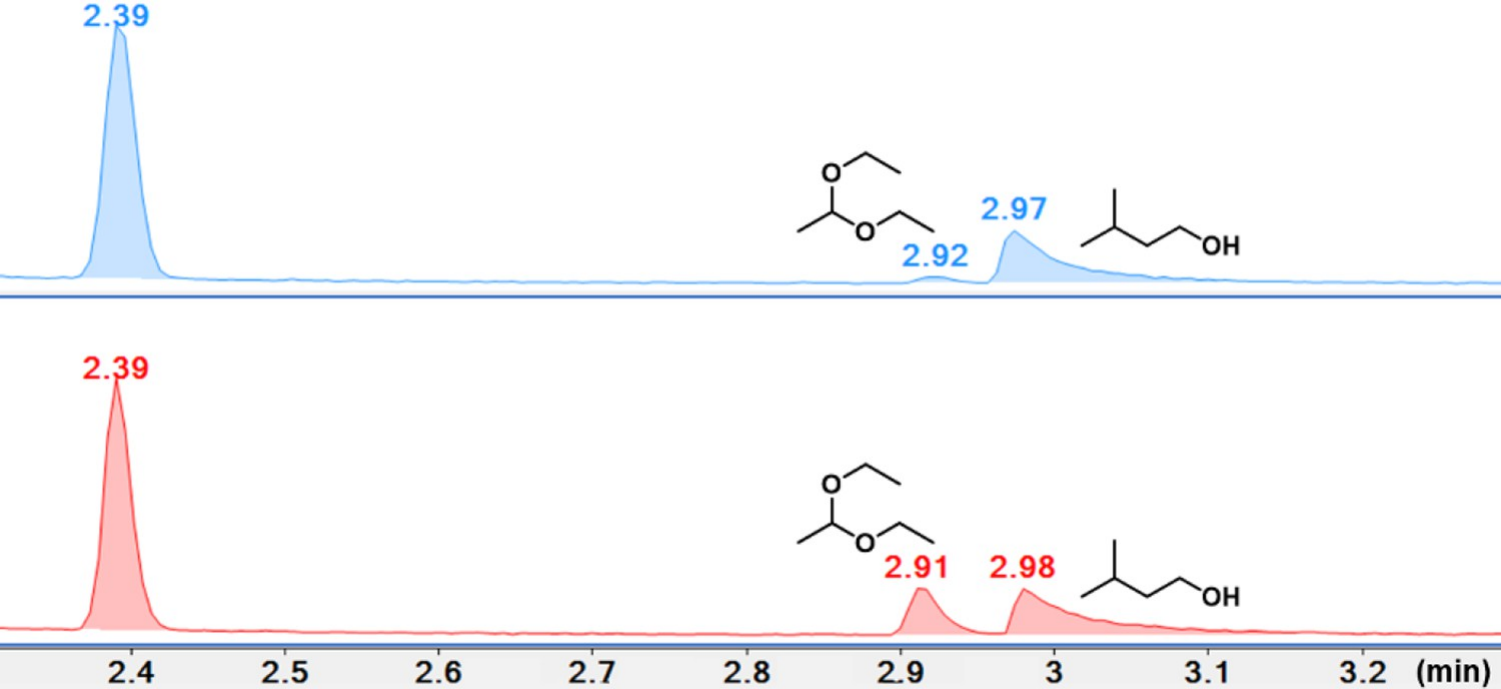
Stacked gas chromatograms from charcoal extracts of the wild-type (blue) and the mutant cultures (red). Cyclohexene was used as an internal reference (retention time = 2.39 minutes). The mutant samples exhibited roughly ten times greater amounts of acetal (retention time = 2.91–2.92 minutes) than the wild-type.

**Fig 10:**
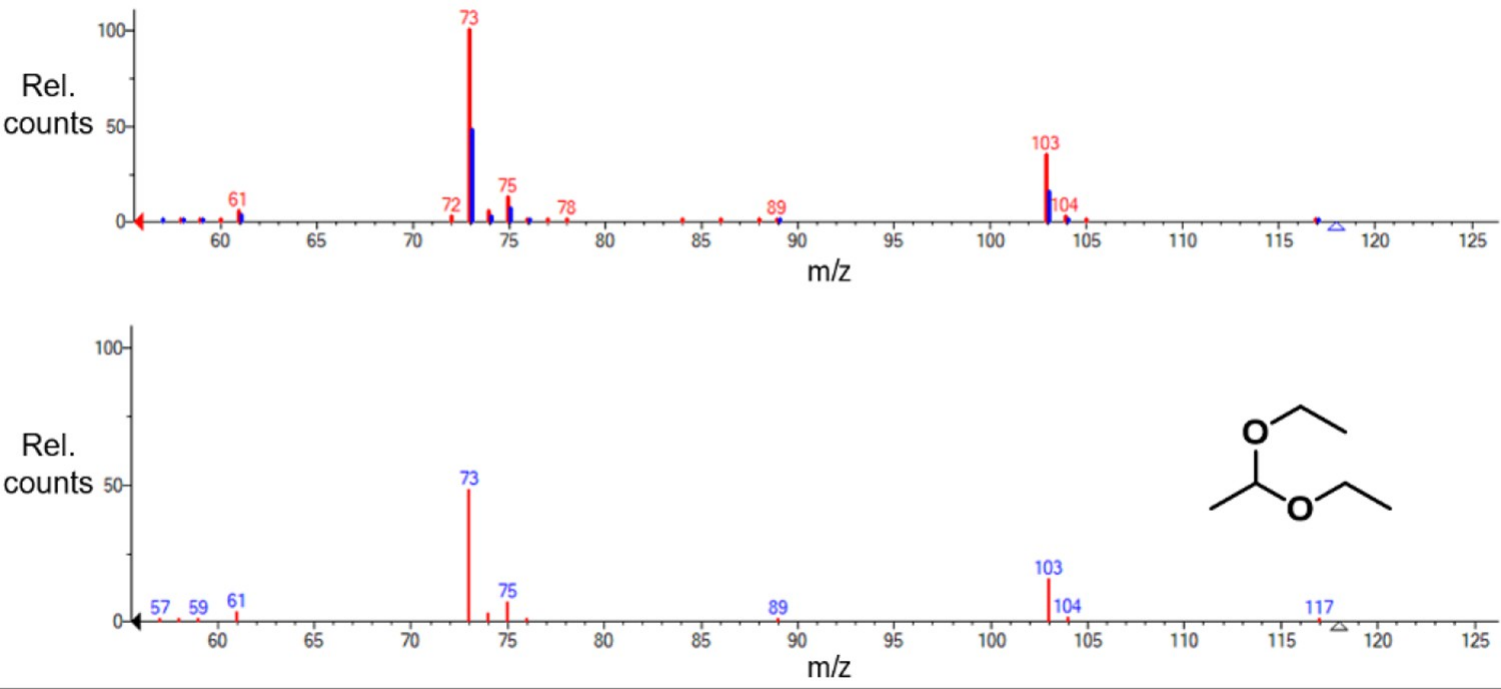
Identification of acetal. Top: Experimentally obtained mass spectrum of acetal (red) overlayed with the NIST reference standard for acetal (blue). Bottom: Reference standard mass spectrum for acetal.

In the organic extracts obtained from the charcoal plugs, we found two major components in both the wild-type and mutant samples. The wild-type sample contained isoamyl alcohol (retention time = 2.97–2.98 minutes), and traces of acetal (retention time = 2.91–2.92 minutes). The mutant sample contained similar amounts of isoamyl alcohol, but acetal was present in roughly ten times greater amount as compared to the wild-type sample (Fig 9). The mass spectrum of acetal was in complete agreement with the reference MS provided by NIST (Fig 10).

### Acetal promotes cell death in the presence of nucleosides and nucleobases

**Fig 11:**
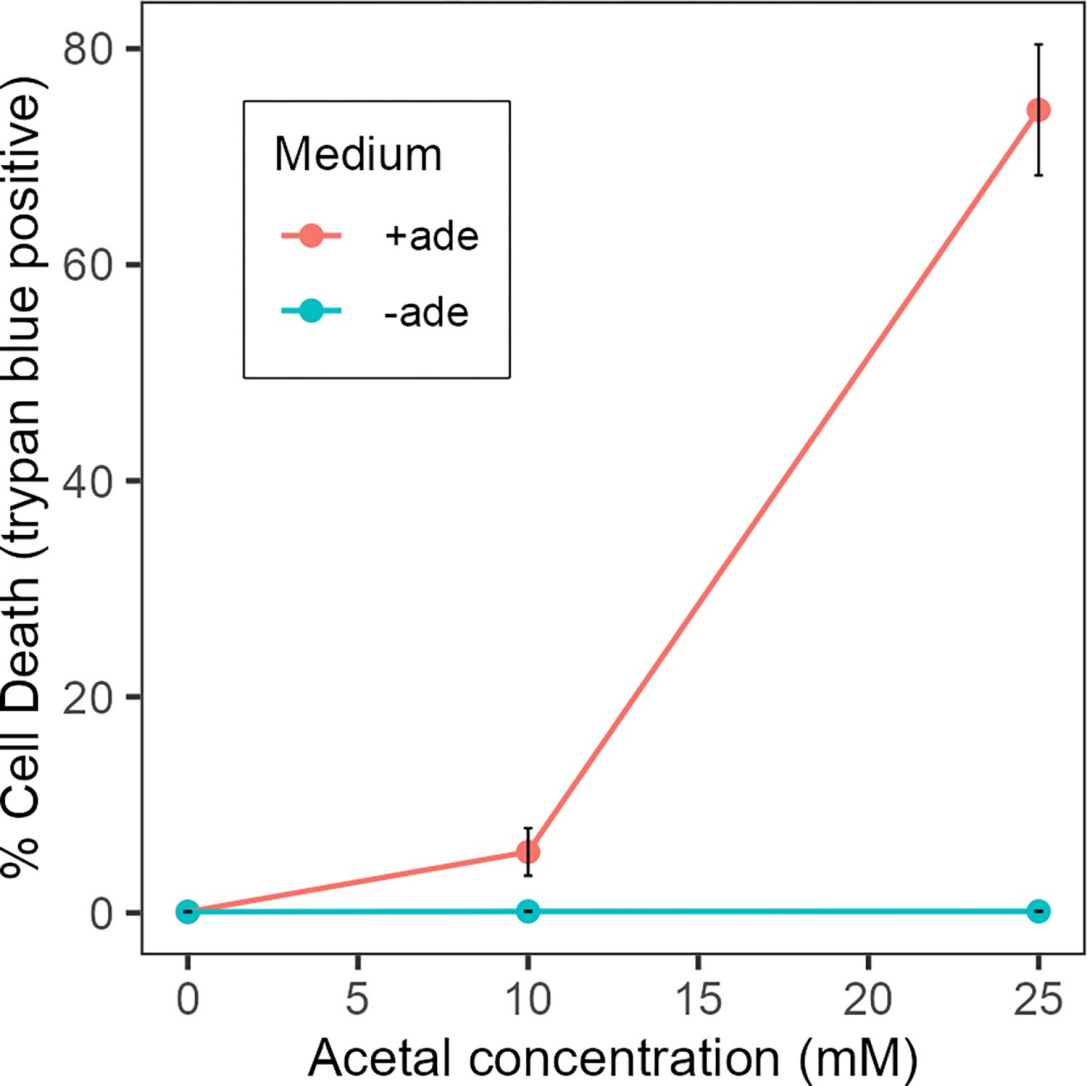
Acetal promotes purine-dependent cell death. SC media with or without 10 mM adenine was supplemented with the indicated concentrations of acetal, inoculated with log-phase SC5314 cells (5 x 10^6^ cells/ml) and assessed by trypan blue staining after 7 h. Each point indicates the average of three independent determinations. Crossbars indicate standard error of the mean of three independent determinations.

As the presence of tetramethylurea and an increased amount of acetal were unique to the *nuo3Δ/Δ* mutant both were tested for their ability to stimulate purine-dependent cell death. Each compound was added to SC media supplemented with 10 mM adenine at concentrations ranging from 100 μM to 25 mM. Tetramethylurea at concentrations ranging from 1 to 20 mM had no effect on viability in the presence of 10 mM adenine. Acetal, however, promoted purine-dependent cell death. As shown in Fig 11, acetal addition alone had no effect on cell viability. When combined with 10 mM adenine, there was substantial loss of cell viability dependent on acetal concentration. Thus, acetal appears to be at least one volatile compound produced by the *nuo3Δ/Δ* mutant which acts synergistically with purines to promote cell death of *C*. *albicans* cells.

## Discussion

This study identified a unique and previously unknown synergistic and toxic metabolic interaction between acetal and purines. This effect is surprising in light of current uses of acetal. Acetal is a GRAS (generally regarded as safe) compound used as a food additive in baked goods, beverages, chewing gum, frozen dairy and other food items [41, 42]. It is used in perfumery and production of pharmaceuticals [43]. It is the major acetal in sherry wines to which it contributes fruity notes [44, 45] and is produced during beer fermentation with lager yeast [46]. No negative or toxic effects have been reported for this compound emphasizing the novelty of the current findings.

GC-MS analysis identified a number of volatile compounds all of which were common to both the respiratory mutant and wild-type cells. The exception was tetramethylurea which was only found in the mutant cultures. However, acetal levels were increased approximately 10-fold in cultures of the respiratory mutants. The increased acetal production is likely related to altered carbon metabolism in the mutant. Acetal is formed in a reversible, acid catalyzed reaction between acetaldehyde and ethanol [47–49]. Since respiration is decreased about 70% in Complex I deficient cells [32], these cells must rely more heavily on fermentation to maintain energy levels. Consequently, the mutant cells produced significantly more ethanol than wild-type cells (Table 2) which would drive acetal formation. Acetal alone had no apparent negative effects as evidenced by the normal growth rate and retention of viability when the mutant was cultured in SC medium (Fig 2), conditions in which acetal was shown to accumulate (Fig 6), or in SC medium supplemented with acetal (Fig 8). The lethal effect of acetal was manifested only in the presence of purine nucleosides or nucleobases.

The media-conditional nature of the lethal phenotype is apparently related to differences in content of nucleotides, nucleosides and nucleobases. Both SC and YPD contain a full complement of amino acids and lipid extraction failed to alter the lethal consequence of growth in YPD, suggesting that neither amino acids nor lipids are involved. Fractionation of BactoPeptone by size exclusion chromatography demonstrated a broad range of cell death stimulating molecules (Fig 7). Importantly, the delayed elution of multiple stimulatory peaks is consistent with the known adsorption behavior of nucleotides, nucleotides and bases on acrylamide-based chromatography media [35–37, 50]. The diverse size range of stimulatory molecules is consistent with a pancreatic digest of animal tissue. The pancreas produces DNaseI and RNase A. DNase I hydrolyzes DNA to a mixture of mono-, di- and tetranucleotides, with some higher order oligonucleotides [51, 52]. RNase A cleaves specifically after pyrimidine residues producing a mixture of oligoribonucleotides [53]. Together these hydrolases would generate a diversity of products consistent with the observed elution pattern. As yeast extract also stimulated cell death, it should be noted that yeast extract, an autolysate of yeast, also contains nucleic acids and their digestion products [54, 55]. These elution profile characteristics led to a presumptive identification of the active components as nucleo-compounds, a conclusion supported by the lethality promoting effects of purified ribosides, deoxy-ribosides and bases (Fig 8). However, given the complexity of BactoPeptone and the observed elution profile, the potential involvement of other digestion products or metabolites cannot be excluded. It should also be noted that the SC media contains a low level of adenine (0.14 mM) which was fully consumed by early stationary phase as judged by the inability of conditioned SC medium to support growth of an *ade2Δ/Δ* mutant (<0.5 generations after 24 h) [56].

The lethal effect was promoted by the purine nucleosides deoxyguanosine, deoxyadenosine, guanosine, adenosine and inosine, as well as the nucleobases adenine, hypoxanthine and xanthine (Fig 8). Guanine was not tested due to its extremely low solubility. Nucleotides were also not tested as *C albicans* dephosphorylates extracellular nucleotides to nucleosides [57]. Notably, cytidine, uridine, cytosine and uracil were without effect. Differential uptake is unlikely to account for this dichotomy as *C*. *albicans* contains cytosine and uridine permeases [58], several putative purine-cytosine permeases [59] and a concentrative H^+^-nucleoside symporter that can transport uridine [60, 61]. This points to purines specifically as effectors of the phenotype. Furthermore, the observation that uric acid, the initial product in purine catabolism [39], had no effect on cell viability points to the involvement of the purine salvage pathways. *C*. *albicans* contains a complete purine salvage pathway such that all purines are interconvertible [39]. Thus, it is unclear whether the lethal effects are associated with a particular purine, though adenosine and hypoxanthine were the most stimulatory, followed by adenine (Fig 8A).

The molecular mechanism(s) underlying the synergistic lethal interaction of acetal and purines remains to be identified and the unprecedented nature of the observation does not suggest obvious candidates. Loss of viability initiates at late-log/early stationary phase of growth. This presumably reflects the time at which acetal has accumulated to the necessary concentration, but quantitative assessment is necessary to establish this. Since the kinetics of cell death are similar for log-phase wild-type cells or mutant cells suspended in conditioned YPD (Fig 5), the lethal effect is not related to the stage of cell growth or respiratory capacity of the cells. It is also apparent that the process involves a period of cell growth and metabolism (Fig 5).

An interesting facet of the phenotype is its positive correlation with purine concentration (Fig 8). The higher the external adenine concentration, the more extensive the cell death, suggesting adenine is being directed into a toxic metabolite. Chemically modified purines have a long history of clinical use based on their ability to inhibit RNA and DNA metabolism [62, 63]. A simple explanation of the present observations is in situ chemical modification of purines by acetal resulting in a toxic product. This is unlikely, however, as acetals have limited chemical reactivity which favors them as protecting groups in organic synthesis. A more likely scenario is disruption of normal control of purine metabolism leading to accumulation of one or more toxic intermediates. As purine metabolism impinges on most every aspect of cell growth and reproduction, there are multiple possibilities.

A noteworthy feature of the phenotype reported here is that the synergy of acetal with purines leads to membrane disruption and cell death. Supplementation of bacterial growth media with adenine causes growth inhibition due to depletion of guanine nucleotide pools [64], inhibition of folic acid co-enzymes [65], or suppression of thiamine biosynthesis [66], but bactericidal effects were not reported. Similarly, adenine and adenosine are cytostatic for human lymphocytes [67]. In contrast, *C*. *albicans* is uninhibited by external adenine or adenosine as high as 10 mM in concentration indicating the cells maintain full control of purine metabolism under these conditions. In the presence of millimolar concentrations *C*. *albicans* will accumulate adenine, adenosine or xanthine within the vacuole [68]. Presumably this allows storage for future use and regulates cytoplasmic concentrations of purines. Disruption of this control is one possible target of acetal toxicity. At the same time, adenine availability suppresses activation of the purine biosynthetic pathway via the transcription factors Grf10 and Bas1 [69], which also control one-carbon metabolism and phosphate, copper and iron responses [70]. A related control system exists in *S*. *cerevisiae* where an intermediate of the adenine biosynthetic pathway, ZMP, controls NAD^+^ biosynthesis as well [71]. While acetal may disrupt any of these regulatory controls, it should be noted that extensive genetic manipulations of these control and biosynthetic pathways in yeast has not uncovered a comparable adenine-dependent cell death phenotype. In this regard, it should be noted that ZMP accumulation is toxic and inhibits cell growth [72] and other purine related metabolic intermediates, such as NAD(P)X, (R,S)-AdoMet, or 5′-methylthioadenosine can inhibit growth [21, 28]. But again, these are only growth inhibitory. It is the dramatic and rapid loss of cell viability and integrity that distinguishes the synergetic interaction of acetal and purines and prompts further investigation to understand the molecular basis of this phenotype and its potential application to antifungal drug development.

## Supporting information

S1 Fig Cell death kinetics of *nuo3Δ/Δ* mutant in conditioned YPD media. Cells of strain mt1179.41 (*nuo3Δ/Δ*) were collected in log-phase and suspended at a density of 5 x 10^6^ cells/ml in YPD media conditioned by prior growth of strain mt1179.41 or strain SC5314. Samples were withdrawn at the indicated time points and assessed by trypan blue staining and microscopic count. Points represent the average and cross-bars the standard error of the mean of three independent determinations.
(TIF)

S1 Table Effect of various peptones on cell death. (XLSX)

S2 Table Effect of nucleosides and bases in purged conditioned SC media. (XLSX)

S1 Dataset Colony and cell counts. (XLSX)
