# Supplementary figures and images for: Lethal metabolism of Candida albicans respiratory mutants
Source: PLoS One. 2024 Apr 5;19(4):e0300630. doi: 10.1371/journal.pone.0300630 (PMC10997084; doi:10.1371/journal.pone.0300630)

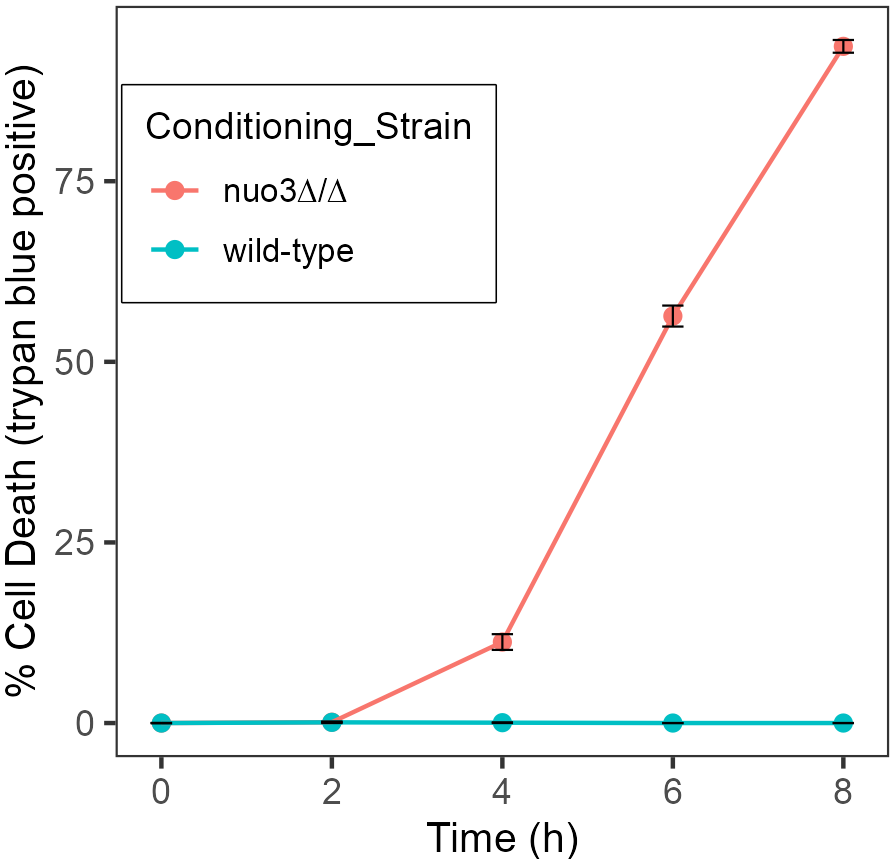

Supplement: S1 Fig — Cells of strain mt1179.41 (nuo3Δ/Δ) were collected in log-phase and suspended at a density of 5 x 10^6^ cells/ml in YPD media conditioned by prior growth of strain mt1179.41 or strain SC5314. Samples were withdrawn at the indicated time points and assessed by trypan blue staining and microscopic count. Points represent the average and cross-bars the standard error of the mean of three independent determinations. (TIF) [file pone.0300630.s001.tif]
